# Highway Safety with an Intelligent Headlight System for Improved Nighttime Driving

**DOI:** 10.3390/s24227283

**Published:** 2024-11-14

**Authors:** Jacob Kwaku Nkrumah, Yingfeng Cai, Ammar Jafaripournimchahi, Hai Wang, Vincent Akolbire Atindana

**Affiliations:** 1 Automotive Engineering Research Institute, Jiangsu University, Zhenjiang 212013, China; nkrumahjacob6@gmail.com (J.K.N.); jafaripour.a@gmail.com (A.J.); vaatindana@tatu.edu.gh (V.A.A.); 2 School of Automotive and Traffic Engineering, Jiangsu University, Zhenjiang 212013, China; wanghai1019@163.com

**Keywords:** accident prevention, highway safety, high beams, intelligent headlight, machine-learning-based technology, sensor-based technology

## Abstract

Automotive headlights are crucial for nighttime driving, but accidents frequently occur when drivers fail to dim their high beams in the presence of oncoming vehicles, causing temporary blindness and increasing the risk of collisions. To address this problem, the current study developed an intelligent headlight system using a sensor-based approach to control headlight beam intensity. This system is designed to distinguish between various light sources, including streetlights, building lights, and moving vehicle lights. The primary goal of the study was to create an affordable alternative to machine-learning-based intelligent headlight systems, which are limited to high-end vehicles due to the high cost of their components. In simulations, the proposed system achieved a 98% success rate, showing enhanced responsiveness, particularly when detecting an approaching vehicle at 90°. The system’s effectiveness was further validated through real-vehicle implementation, confirming the feasibility of the approach. By automating headlight control, the system reduces driver fatigue, enhances safety, and minimizes nighttime highway accidents, contributing to a safer driving environment.

## 1. Introduction

Headlights are essential components on vehicles whose functions become more pronounced during nighttime and adverse weather conditions while driving. The headlight is equipped with two main light beams, namely the low beam and the high beam. The low beam is employed to illuminate close and mid-range distances in front of the vehicle, offering the driver clearer visibility at short and medium ranges. It features an asymmetrical luminous intensity distribution pattern, covering approximately 100 m [1,2,3]. Conversely, the high beam is utilized for long-distance illumination when no oncoming vehicles are present. It exhibits a symmetrical luminous intensity distribution pattern and can illuminate distances beyond 200 m [4,5]. However, the conventional headlight system remains manual, relying on the driver to switch between high and low beams depending on road conditions. Unfortunately, this discretion often leads to misuse and abuse, thereby posing additional risks on the roads. To address these challenges and bolster road safety, intelligent headlights represent a promising alternative. An intelligent headlight system autonomously adjusts the headlight beams without explicit driver intervention [6,7,8].

Intelligent transportation systems have emerged as a dominant force in the automotive industry, fundamentally transforming traditional operations. These systems are primarily designed to augment vehicle safety and enhance ride comfort. Driving a vehicle is primarily a visual task, with approximately 90% of the information gathered by drivers being visual [9,10,11]. Automating headlight beam adjustments and incorporating advanced technologies can help mitigate glare-related accidents and create a safer driving environment for all road users. Drivers’ intermittent switching of headlight beams can lead to driver fatigue, particularly considering the recent astronomical increase in vehicular population on highways. The increase in vehicular population on highways has placed a higher demand on drivers to frequently switch between high and low beams during nighttime driving due to the elevated traffic levels [12]. Consequently, numerous studies and designs are emerging to automate the control function of changing headlight beams from high to low during nighttime driving, shifting this responsibility from the driver to an electronic system for automatic adjustment. This trend has led to the proposition of various intelligent headlight designs utilizing different luminous intensity control approaches, such as machine-learning-based, fuzzy-logic-based, pulse-width-modulation-based, sensor-based, and many others [13].

Among these approaches, machine-learning-based luminous intensity control is notable but often employs expensive technology, limiting its application primarily to high-end cars and potentially raising vehicle costs beyond the reach of low-income earners. To strike a balance between technological effectiveness and cost, the sensor-based luminous intensity control approach emerges as the most promising. This approach has been widely adopted by many designers for intelligent headlight beam luminous intensity management. The sensor-based control approach typically utilizes photo-sensors such as Light-dependent resistors (LDRs) to detect varying luminous intensity levels in the surrounding road environment. Additionally, ultrasonic sensors are commonly used for distance measurements between vehicles before beam adjustment. Numerous designers have implemented this luminous intensity control method in intelligent headlight systems. For example, authors in [14,15,16] designed an intelligent headlight system employing an LDR, transistor, and relay for effective headlight high and low beam control, enhancing driving safety and visibility. In a similar vein, the authors proposed a headlight luminous intensity control approach based on dual-pixel Active Pixel Sensor (APS) architecture for vision-based speed measurement applications [17]. This sensor integrates two types of imaging elements to generate two coherent images for vehicle identification and speed estimation, demonstrating robust performance. Furthermore, authors in [18] designed an intelligent headlight system for accident avoidance, integrating various sensors such as LDR, Doppler radar, optical fog sensor, Video Image Process (VIP) sensor, and ultrasonic sensor into an Arduino microcontroller, offering a more affordable alternative compared to existing luxury car systems. Ajithkumar et al. [19] utilized Arduino microcontrollers, LDR sensors, and ultrasonic sensors to develop Arduino-based smart headlight systems, effectively automating headlight beam adjustment and reducing glare effects on opposing drivers.

Many authors, including those in [20,21,22,23,24], employed Arduino microcontrollers, LDR sensors, and ultrasonic sensors with the aim of designing an adaptive headlight system that automatically varies the beams depending on the surrounding lighting conditions. Their design succeeded by eliminating the driver from controlling the headlight beams and also reducing the effects of the headlight’s high beam on the opposite vehicle’s driver. Furthermore, authors in [25,26] designed an advanced headlight control system capable of automatically adjusting headlight beams horizontally to illuminate curved roads, employing steering wheel output to drive the headlights left or right based on steering wheel rotation. These innovative designs and approaches demonstrate the ongoing efforts to enhance road safety through intelligent headlight systems, highlighting the importance of automated beam adjustment in improving driving conditions and reducing accidents. Road accidents are a serious concern and glare from headlights’ high beams during nighttime driving poses a significant challenge for drivers, often leading to accidents. Manually controlling headlight beams becomes particularly burdensome for drivers when oncoming vehicles are within 150 m. Therefore, it is imperative to install a safety control system in vehicles that automatically dims the headlights’ high beam when excessive luminous intensity is detected, reducing glare and enhancing safety [27].

The contribution of this study is to improve highway safety by reducing accidents caused by headlight high beam glare. It proposes a more affordable alternative to machine learning-based intelligent headlight systems, which are typically expensive and limited to high-end cars. By making this technology accessible to all vehicle levels, the study promotes its use in low- and middle-income countries, where nighttime and single-lane road accidents are common. The intelligent headlight system integrates a radar sensor, replacing the traditional ultrasonic sensor, to measure both the distance and speed of approaching vehicles. This enhances the system’s ability to distinguish between moving and stationary lights, improving overall safety. Additionally, the system includes an alert device (buzzer) to notify the driver when the headlight switches from a high beam to a low beam, indicating an approaching vehicle. This feature ensures the driver’s attention remains on the road during critical moments, reducing the risk of head-on collisions. Recognizing that the driver is ultimately responsible for vehicle control, the study highlights the importance of keeping the driver informed and involved, making the system more user-friendly and effective.

## 2. Methods and Materials

The proposed approach utilizes basic yet efficient electronic components for its operation. The selection of these components aims to strike a balance between technological sophistication and cost-effectiveness. This decision was made considering several factors, including the simplicity of the architecture, robustness against adverse weather conditions, and the widespread use of sensors in various applications, such as smart headlight beam luminous intensity control systems. Furthermore, the extensive use of sensors in vehicle applications over decades without significant issues further reinforces the reliability and effectiveness of this approach. Modern automobiles rely on over 100 sensors to manage various vehicle functions, underscoring the established track record of sensor technology [28,29,30]. For the simulation of the proposed approach, specific sensors were employed, including the light-dependent resistor (LDR) sensor, the radar sensor, and a buzzer. Specifically, the radar and LDR sensors were calibrated before their integration into the system. The calibration processes are as follows:

Calibration process for radar sensor

1. The radar sensor was calibrated by initially installing the radar sensor in the engine of the intelligent headlight-enabled vehicle; it was located behind the radiator, ensuring that it had an unobstructed view of the road ahead.
2. A test was performed while the intelligent headlight-enabled vehicle was in static condition in a controlled environment where known distances and objects were placed at various points in front of the sensor.
3. The recorded radar readings were compared to the actual distances to create a baseline measurement.
4. The intelligent headlight-enabled vehicle was driven on live roads and at various distances from stationary to determine if the sensor could detect moving objects.
5. The data collected was analyzed to identify any discrepancies in detection and response time.
6. The sensor settings, such as gain, frequency, and range, were fine-tuned based on the data analysis.

Calibration Process for the LDR Sensor

1. The LDR sensor was placed on the dashboard, and its light-sensitive part was made to face the road for effective measurement of the headlight beams of the oncoming vehicles, ideally away from direct interference.
2. The resistance of the LDR sensor was measured at different luminous intensities to understand the characteristics of the sensor.
3. The LDR sensor was tested in real-world lighting conditions, such as bright daylight, dusk, and nighttime, to determine its functionality.

**Figure 1:**
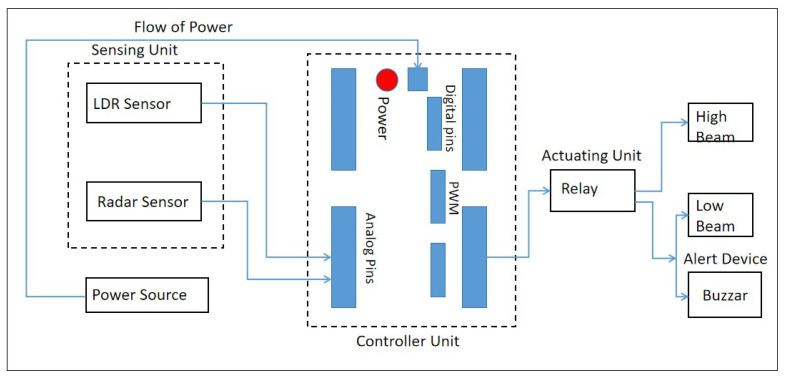
Block Diagram of the Proposed Intelligent Headlight System.

Figure 1 illustrates the block diagram of the proposed intelligent headlight system, representing the culmination of the chosen sensor-based approach for this research. This system aims to enhance headlight performance, adaptability, and driver safety during nighttime driving conditions.

### 2.1. Description of Materials

To achieve the design objectives of the proposed intelligent headlight beam luminous intensity control system, several critical components were selected for their specific functionalities and compatibility with the system. Each material plays a distinct role in ensuring the effective operation and responsiveness of the headlight control system. These materials include the Arduino microcontroller, a Single Pole Double Throw (SPDT) relay, an LDR (Light Dependent Resistor) sensor, an active buzzer, and a radar sensor.

First, the Arduino microcontroller serves as the central processing unit for the system, facilitating communication and control across various components. With 14 digital I/O pins (six of which support Pulse Width Modulation (PWM)) and six analog input pins, this microcontroller is highly adaptable for intricate control tasks. Additionally, it includes a 16 MHz ceramic resonator, USB connectivity, a power jack, an In-Circuit Serial Programming (ICSP) header, and a reset button, operating within a voltage range of 5 V. These features make it an ideal choice for managing the signal processing and control functions necessary for the headlight system.

Next, the Single Pole Double Throw (SPDT) relay was chosen for its reliability and compatibility with the Arduino microcontroller. This relay enables the control of high-voltage components, such as the headlights, through the Arduino’s low-voltage signal output. Its robust design allows for efficient switching between high and low beam intensities, contributing to the system’s overall reliability and precision.

The LDR sensor (Light Dependent Resistor) is essential for detecting ambient light levels and adjusting the luminous intensity of the headlights accordingly. The LDR’s resistance varies inversely with the incident light intensity, a phenomenon known as photoconductivity. As the surrounding light increases, the LDR’s resistance decreases, allowing the system to sense changes in ambient light and adjust headlight output automatically.

An active buzzer was incorporated for its ease of integration and ability to produce louder sounds compared to passive buzzers. This component serves as an alert mechanism, notifying the driver when the headlight output switches from high to low beams, particularly in response to detected oncoming high beams. Its inclusion enhances driver awareness, making the transition between beam intensities more apparent.

Finally, the radar sensor was chosen for its ability to detect movement and measure distance under various environmental conditions. Unlike optical sensors, radar sensors are capable of penetrating materials such as plastic, wood, and drywall, which makes them suitable for through-wall applications and resilient in adverse weather conditions. The Delphi radar sensor, specifically, was selected for its wide field of view and extended horizontal range, detecting objects up to approximately 174 m. This capability ensures that the system can identify oncoming vehicles in time to adjust the headlights, improving safety and responsiveness.

### 2.2. Reference Luminous Intensity

**Figure 2:**
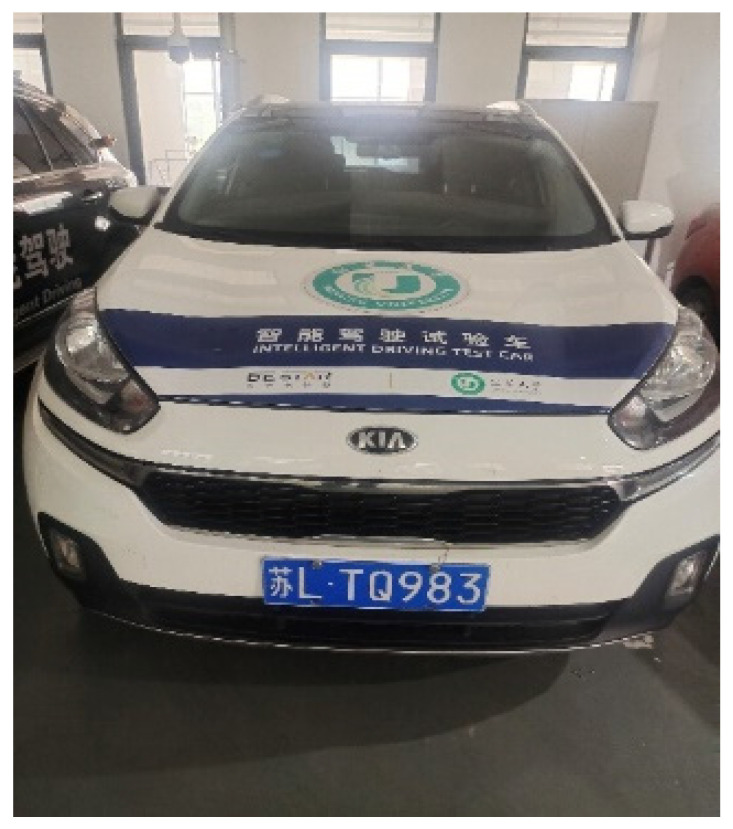
The vehicle employed for the experiment.

When designing an intelligent headlight system using sensor-based technology, obtaining accurate reference luminous intensities for programming the controller is essential. These reference values can be determined by measuring the luminous intensities produced by various light sources in different headlight designs. To achieve this, a test was conducted using the vehicle shown in Figure 2. Three different headlight light sources, namely, Halogen, HID, and LED, were sequentially installed in each headlight, and their luminous intensities were measured using a photometer. To ensure precise measurement of each light source’s true luminous intensity, the testing was conducted in a sufficiently dark environment. The luminous intensities of each light source were measured individually in both projector and reflector headlights at various distances.

#### Luminous Intensity Models

Determining the reference luminous intensity is a critical prerequisite in designing an efficient, intelligent headlight controller. This reference luminous intensity serves as the programmed benchmark for the controller to detect headlight beams emanating from various light sources and headlight types. To effectively establish reference luminous intensities adaptable to all light sources and headlight types, the following models may be employed:

(1) $$l_{b}m_{\mathrm{int}}(R)=\frac{\sum_{s=n}^{i=n}i_{s_{1}}+i_{s_{2}}+i_{s_{3}}+\ldots \ldots i_{s_{n}}}{n_{s}}$$

(2) $$l_{b}m_{\mathrm{int}}(P)=\frac{\sum_{s=n}^{i=n}i_{s_{1}}+i_{s_{2}}+i_{s_{3}}+\ldots \ldots i_{s_{n}}}{n_{s}}$$

(3) $$R_{\mathrm{int}}l_{b}=\frac{\sum l_{b}m_{\mathrm{int}}(R)+l_{b}m_{\mathrm{int}}(P)+\ldots l_{b}m_{\mathrm{int}}(n)}{n_{h}}$$

where, $l_{b}m_{\mathrm{int}}(R)$ is the low beam mean intensity in a reflector headlight, $l_{b}m_{\mathrm{int}}(P)$ is the low beam mean intensity in a projector headlight, $R_{\mathrm{int}}l_{b}$ is the mean of means or reference luminous intensity of the low beam, $i_{s_{1}},i_{s_{2}},i_{s_{3}},\ldots \ldots i_{s_{n}}$ are the beam luminous intensities of different light sources (e.g., Halogen, HID, and LED), $n_{s}$ is the number of light sources, and $n_{h}$ is the number of headlight types. The model Equation (3) then becomes the equation for determining the reference luminous intensity for the low beam. In the same vein, the reference luminous intensity of the high beam can be determined as follows:

(4) $$h_{b}m_{\mathrm{int}}(R)=\frac{\sum_{s=n}^{i=n}i_{s_{1}}+i_{s_{2}}+i_{s_{3}}+\ldots \ldots i_{s_{n}}}{n_{s}}$$

(5) $$h_{b}m_{\mathrm{int}}(P)=\frac{\sum_{s=n}^{i=n}i_{s_{1}}+i_{s_{2}}+i_{s_{3}}+\ldots \ldots i_{s_{n}}}{n_{s}}$$

(6) $$R_{\mathrm{int}}h_{b}=\frac{\sum h_{b}m_{\mathrm{int}}(R)+h_{b}m_{\mathrm{int}}(P)+\ldots h_{b}m_{\mathrm{int}}(n)}{n_{h}}$$

where $h_{b}m_{\mathrm{int}}(R)$ is the high beam mean luminous intensity in a reflector headlight, $h_{b}m_{\mathrm{int}}(P)$ is the high beam mean luminous intensity in a projector headlight, $R_{\mathrm{int}}h_{b}$ is the mean of means or reference luminous intensity of the high beam. Equation (6) then becomes the equation for determining the reference luminous intensity for the high beam. The reference luminous intensities for both the high and low beams are determined when the mean intensities of the projector and reflector headlights converge. The luminous intensity at the point of convergence serves as the reference luminous intensity and, when incorporated into the controller design, ensures adaptability to all light sources.

### 2.3. The Proposed Intelligent Headlight Design Algorithm

The proposed intelligent headlight operates according to a specific algorithm, outlining the step-by-step procedure and logical order in which the Arduino microcontroller controls the luminous intensities of the headlight beams. The system functions by comparing the output signals from both the light-dependent resistor (LDR) and the radar sensor with the output voltage threshold set in the processor. This threshold determines whether the headlight output should switch from a high beam to a low beam or vice versa. The Arduino microcontroller is programmed to send a voltage signal to each of the beams one at a time, depending on which beam’s threshold is met. The output signals from the Arduino microcontroller are labeled as ‘HIGH’ and ‘LOW’. When the Arduino microcontroller sends a ‘HIGH’ voltage signal to the relay, it indicates that the relay should switch the beam from the high beam to the low beam. Conversely, when the ‘LOW’ voltage signal below the threshold is sent, it suggests that the relay should either maintain the high beam or switch from the low beam to the high beam. To achieve the desired outcome, the Arduino microcontroller was programmed using Integrated Development Environment (IDE) software based on the following algorithms:

#### Simplified Control Algorithms

The intelligent headlight system employed an algorithm (Algorithm 1) that uses defined thresholds for vehicle speed (>0 m/s) and luminous intensity (>400 lux). The intelligent headlight system was designed to start in the high beam state. The controller continuously reads data from the radar and LDR sensors. The controller was programmed to check if both the vehicle speed is greater than 0 m/s (>0 m/s) and luminous intensity is greater than 400 lux (>400 lux) in the simulation environment and (>16,000 lux) in the practical implementation phase, respectively. If both conditions are true and the headlights are currently on a high beam, it switches to a low beam and activates the buzzer. If the conditions are not met and the headlights are currently on low beam, it switches back to high beam and deactivates the buzzer. The process repeats indefinitely with a slight delay (100 milliseconds) to ensure continuous monitoring.
**Algorithm 1.** Simplified AlgorithmObject Threshold Speed = 0 m/s Luminous Intensity Threshold = 400 lux Headlight state = Headlight starts in high beam   IF object speed > Threshold Speed and luminous intensity > Threshold Lux:   IF headlight state == high:      SET headlight state = low      TURN ON BUZZER      SWITCH TO LOW BEAM  ELSE:    IF headlight state == low:      SET headlight state = high      TURN OFF BUZZER      SWITCH TO HIGH BEAM WAIT (100 milliseconds) // Delay for next sensor reading

### 2.4. Operational Principles of the Intelligent Headlight System

The intelligent headlight beam luminous intensity control system is designed to automatically manage headlight settings, reducing driver intervention and enhancing road safety. When activated, this system takes control of the headlight beams from the driver, adjusting the beam settings according to the environment and traffic conditions. By utilizing an LDR (Light Dependent Resistor) sensor and a radar sensor, the system can detect various driving situations and adjust the headlight beams accordingly. The LDR sensor is employed to detect the light intensity of oncoming vehicles’ headlights. This sensor transmits an analog signal to the microcontroller, enabling it to recognize changes in light levels. Additionally, a radar sensor is used to measure the speed of oncoming vehicles, which also sends a corresponding signal to the microcontroller. Both sensors work together to provide real-time data about oncoming traffic, allowing the system to make informed decisions on beam intensity adjustments.

In the system’s simulation phase, the microcontroller was programmed with a reference luminous intensity of 400 lux, while in the practical implementation, this threshold is set at 16,000 lux to account for varying brightness in real-world conditions. These specific values were chosen to achieve a balance between optimal visibility and safe illumination, as some headlights emit low brightness levels onto the road. When the incoming beam intensity or speed of an approaching vehicle reaches this threshold, the microcontroller processes the signals from both sensors. If the combined values indicate a high-luminosity source from an oncoming vehicle, the microcontroller initiates a switch from high to low beam. The switching mechanism is controlled through a single pole double throw (SPDT) relay, which is connected to the microcontroller. When the threshold is met, the microcontroller outputs a high-voltage signal to the relay, disengaging the high-beam circuit and activating the low-beam circuit. This transition also triggers an active buzzer to alert the driver, helping them stay focused on the road and be aware of the system’s adjustment.

**Figure 3:**
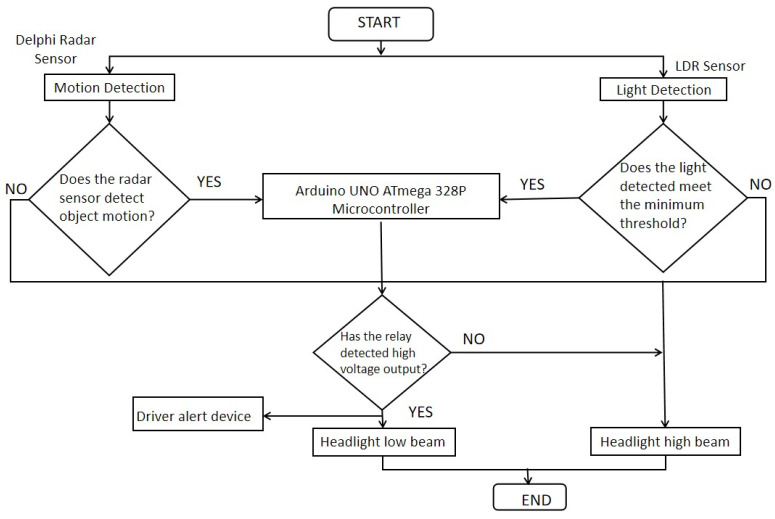
Flow diagram of the intelligent headlight system.

In cases where the LDR sensor detects ambient luminous intensity while the radar sensor does not detect any object movement, the headlight remains in high-beam mode. This differentiation helps avoid unnecessary beam switching in response to static lights, such as streetlights or illuminated buildings. The system’s design ensures that only actual oncoming traffic influences the headlight beam adjustment, preserving high-beam functionality for better road visibility when needed. Power for the entire system is supplied by the vehicle’s 12 V battery, ensuring reliable operation without additional power sources. The intelligent headlight system’s operational flow is depicted in Figure 3, illustrating each step from sensor input to beam switching. Overall, this design optimizes headlight control based on real-time conditions, enhancing road safety and reducing the risk of glare for oncoming drivers.

## 3. Results and Discussions

**Figure 4:**
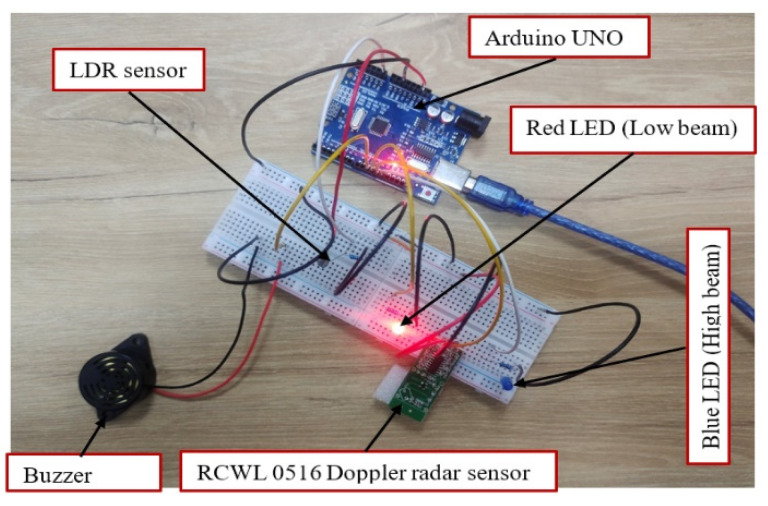
Proposed intelligent headlight low beam.

**Figure 5:**
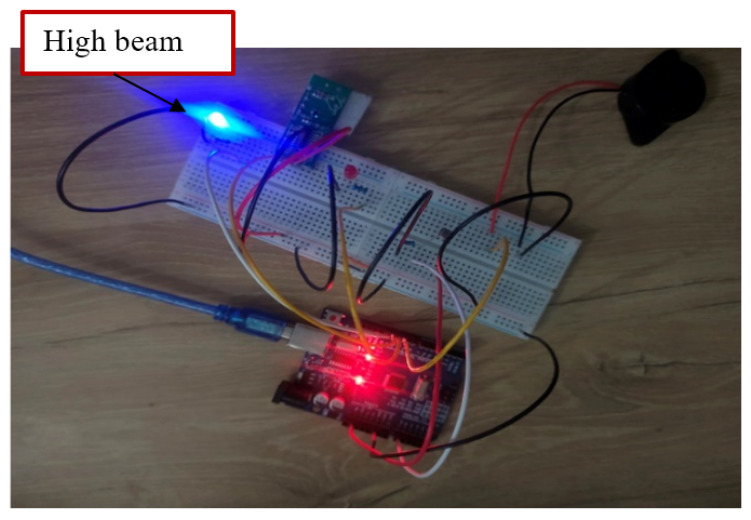
Proposed intelligent headlight high beam.

The primary objective of this study is to design an intelligently discriminative headlight capable of distinguishing between stationary and moving lights. To accomplish this, the study utilized a Radar Continuous Wave Level (RCWL) Doppler sensor, known for detecting object movement within a 7 m range for the simulation. Additionally, a buzzer was integrated into the proposed intelligent headlight system to alert the driver of an approaching vehicle. The simulation setup, depicted in Figure 4, showcased the circuit arrangement of the proposed intelligent headlight, utilizing a red LED to represent the low beam and a blue LED for the high beam. The Arduino microcontroller was responsible for coordinating and comparing the outputs from the attached input devices. In the simulation environment, when the luminous intensity from the opposite direction surpassed the preset threshold luminous intensity and the light source began moving, the headlight output seamlessly switched from a high beam (blue LED) to a low beam (red LED) as shown in Figure 4. Concurrently, the buzzer was triggered with the anticipation of alerting the driver. This confirms the functionality of the proposed intelligent headlight system, and the results obtained are consistent with the results of similar studies conducted by authors in [15,16,18]. The intelligent headlight output remained on the low beam until the light source was extinguished and stopped moving. At this point, the headlight output reverted to a high beam, as illustrated in Figure 5. This successful transition indicated the effective coordination between the LDR sensor and the Doppler radar sensor. This implies that when LDR and radar sensors are utilized in real-world applications for the proposed sensor-based intelligent headlight system, the system is expected to operate as intended. The aim to design and simulate a discriminative intelligent headlight system was accomplished with outstanding results. The key performance indicators (KPIs), such as beam transition from high to low, the incorporation of the alert system, and the discriminatory function by the radar sensor set to assess the functionality of the proposed intelligent headlight, were successfully met, confirming the feasibility and successful operation of the proposed intelligent headlight approach.

The simulated results of the Arduino microcontroller-based intelligent headlight are presented as follows: Initially, the functionality of the Light Dependent Resistor (LDR) sensor was simulated to assess its characteristics and understand the limitations posed by its nonlinearity. Indeed, the nonlinear characteristics of LDRs can present challenges in applications requiring a linear response. To mitigate this, our study partially addressed the nonlinearity limitation of LDRs by employing a segment of the Piecewise Linearization technique. The output of the LDR sensor governed whether the headlight remained in high beam mode or switched to low beam, with the primary objective being to minimize glare from headlight high beams for enhanced nighttime driving safety. In the simulation environment, when the LDR sensor detected a luminous intensity below the reference luminous intensity of the 400-lux threshold set for this simulation. The system maintained the high beam of the headlight, indicating that the road was free; therefore, the driver could use the high beam for extended visibility.

Conversely, when the LDR sensor detected the high beam luminous intensity above the threshold of 400 lux from the opposite direction of the road in the simulation environment, the intelligent headlight-enable car’s headlight output promptly transitioned from the high beam state to the low beam, indicating a successful integration of the LDR into the proposed intelligent headlight system. The envisioned intelligent headlight system maintained the headlight output in the low beam state as long as the LDR sensor continued to detect luminous intensity from the opposite direction. Subsequently, the headlight output reverted to the high beam state when the LDR sensor no longer perceived luminosity from the opposite direction, ensuring illumination for longer distances. These characteristics align with the anticipated behavior of the light-dependent resistor sensor, and the characteristics exhibited by the LDR sensor in this study are consistent with the results obtained by authors in [20,23,24] in a similar study. The functionality of the proposed Arduino microcontroller-based intelligent headlight system in the simulated environment demonstrates the feasibility of the proposed approach for controlling headlight beam luminous intensity using LDR sensors.

**Figure 6:**
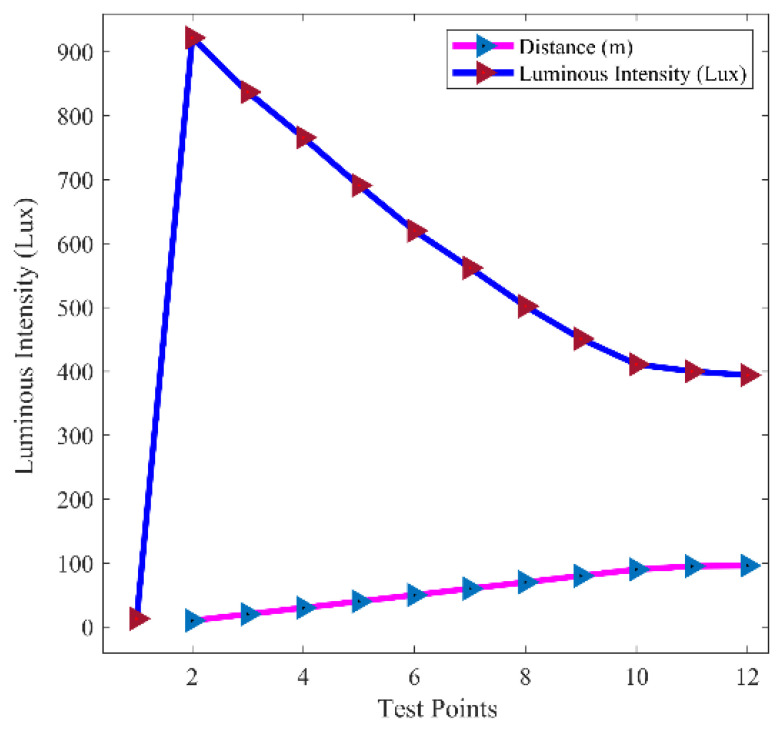
Comparison of LDR light intensities and distances.

The study results of the LDR sensor’s output at different test points were noted to assess the consistency of the sensor at various distances. Measurements of the luminous intensities at different locations along the horizontal distances are illustrated in Figure 6. The relationship depicted in Figure 6 demonstrates an inverse correlation between luminous intensity and distance. It can be observed from Figure 6 that the LDR sensor detected luminous intensities, as measured by the Arduino IDE, decrease as distance increases. The LDR sensor detected a luminous intensity of 13 lux when the environment was completely dark, as registered by the Arduino IDE. This suggests that the ambient illumination of the test environment was 13 lux. At this illumination level, the headlight remained in the high beam state since it did not reach the minimum threshold of 400 lux required to switch the headlight output to the low beam. When the LDR sensor detected a luminous intensity of approximately 922 lux approximately 10 m from the sensor, the headlight output transitioned from the high beam to the low beam state. Particularly, at the 12th test point, when the LDR sensor detected a luminous intensity of about 394 lux at a distance of 96 m, the headlight output reverted from the low beam to the high beam. This transition occurred as the luminous intensity detected by the LDR sensor fell below the minimum threshold required to maintain the headlight output in the low beam state. These observed characteristics demonstrated by the LDR sensor in this simulation align with findings from similar studies conducted by authors in [22,23,24]. These findings indicate consistent behavior in the response of LDR sensors to varying luminous intensities and distances.

**Figure 7:**
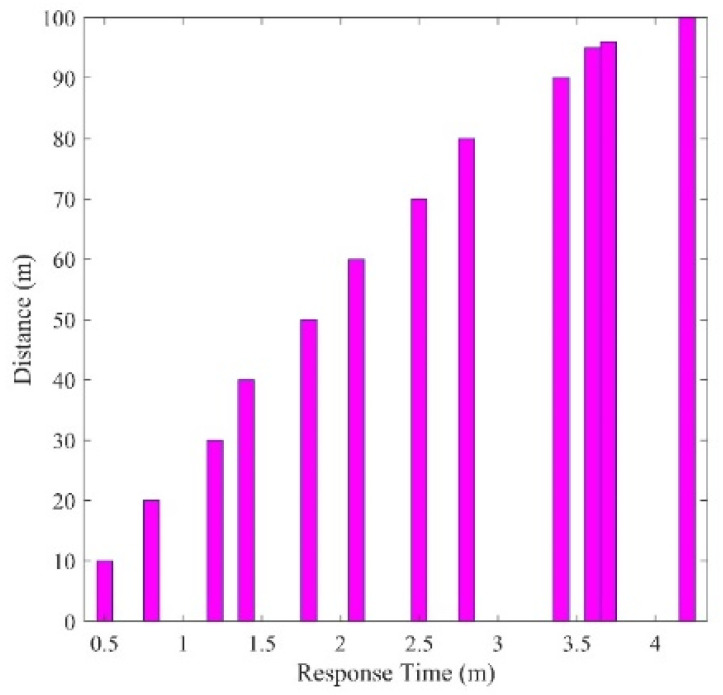
LDR Response Time and Distance.

Figure 7 illustrates the relationship between the response time of the LDR sensor and the distance of the light source. The graph shows a clear trend: as the distance of the light source increases, so does the response time of the LDR sensor. These findings indicate a consistent linear correlation between the LDR sensor’s response time and the distance of the light source at various positions. For instance, when the light source was positioned 10 m from the LDR sensor, it took approximately 0.5 s for the sensor to detect the luminous intensity from the opposite direction. Conversely, at a distance of 100 m, the response time extended to around 4.2 s. This means that at longer distances, the intelligent headlight system would take a long time to cause a transition from the high beam to the low beam as compared to when the oncoming vehicle is closer. The study’s results strongly suggest that the response of the LDR sensor is influenced by the distance of the light source. As the light source moves farther from the sensor, the LDR requires more time to register changes in luminous intensity. This behavior aligns with the expected characteristics of LDRs, which generally exhibit slower responses to light changes when at a distance. The results on response time are consistent with results obtained by authors in [18,20] in a similar study. The consistent linear relationship between response time and distance implies a predictable pattern, which could be advantageous in designing intelligent headlight systems. In a practical highway scenario, vehicles equipped with this technology could adjust their high beam luminous intensity based on their distance from other vehicles. Consequently, when detecting an approaching vehicle’s high beam from a shorter distance, the intelligent headlight system would react more swiftly, thereby reducing glare and enhancing overall road safety. Figure 7 clearly depicts the correlation between distance and the LDR sensor’s response time.

**Figure 8:**
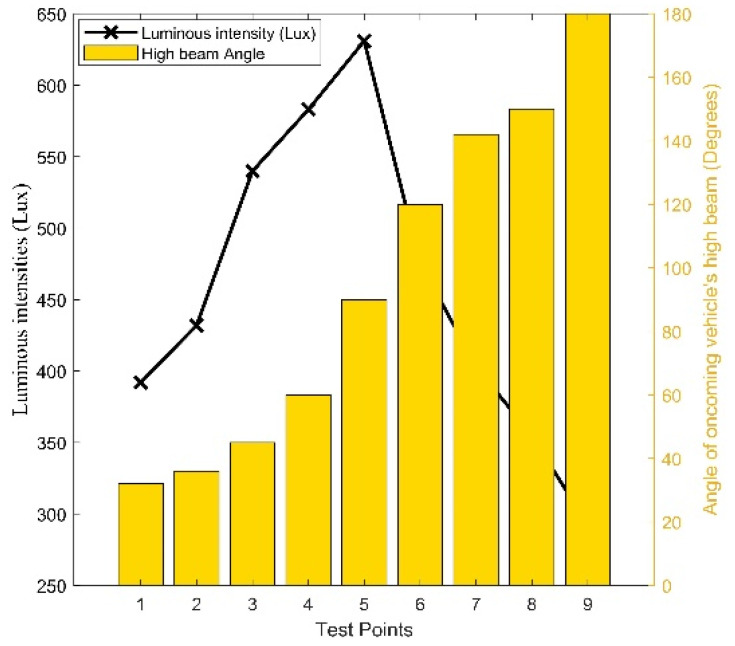
The angle of high beam luminous intensity.

Similarly, the angle of the headlight high beam to the LDR sensor was explored. It was observed that the angle of the high beam significantly impacted the LDR sensor’s output response. Figure 8 presents the correlation between the angle of the high beam and the luminous intensity detected by the LDR sensor. Maintaining a consistent distance of 50 m from the LDR sensor, luminous intensities were measured at nine (9) different angle positions to assess the impact of the angle position of the high beam on the LDR sensor output. The simulation yielded intriguing results, as shown in Figure 8. At an angle position of 32 degrees, the LDR sensor detected a luminous intensity of 392 Lux, which was below the threshold required to trigger a shift from high beam to low beam, thus keeping the headlight output in the high beam state. As the angle position was changed to 36 degrees in the simulation environment, the LDR sensor detected a luminous intensity of 432 Lux, slightly surpassing the reference luminous intensity, prompting the headlight output to transition from high beam to low beam. Subsequent angle positions from 36 to 142 degrees all detected luminous intensities above the set threshold, causing the headlight output to switch to a low beam. At an angle position of 150 degrees, the LDR sensor detected a luminous intensity of 355 Lux, below the required reference luminous intensity.

Accordingly, at this angle position, the headlight output reverted from the low beam to the high beam. However, at an angle position of 180 degrees, the detected luminous intensity was insufficient to prompt the headlight output to transition from the high beam to the low beam, thus keeping it in the high beam state. The findings indicate that the proposed Arduino microcontroller-based intelligent headlight system can detect high beams from angle positions ranging from 36 to 142 degrees in front of the proposed intelligent headlight-enabled vehicle. This range corresponds with the findings of authors in studies [31,32,33], who established that the maximum spread angle of high-beam headlights is 135 degrees. This aligns with measurements taken at various angle positions of the high beam, which indicated a spread angle between 36 and 142 degrees. Of particular note is the angle position of 90 degrees corresponding to test point 5, where the LDR sensor recorded the maximum luminous intensity of 631 Lux. This suggests that vehicles approaching from a 90-degree trajectory to the proposed intelligent headlight would be swiftly detected by the LDR sensor compared to vehicles at other angle positions. Figure 8 demonstrates a complex relationship between the angle of the high beam and the corresponding luminous intensity detection by the LDR sensor. This revelation is valuable for designing intelligent headlight systems employing LDR sensors.

**Figure 9:**
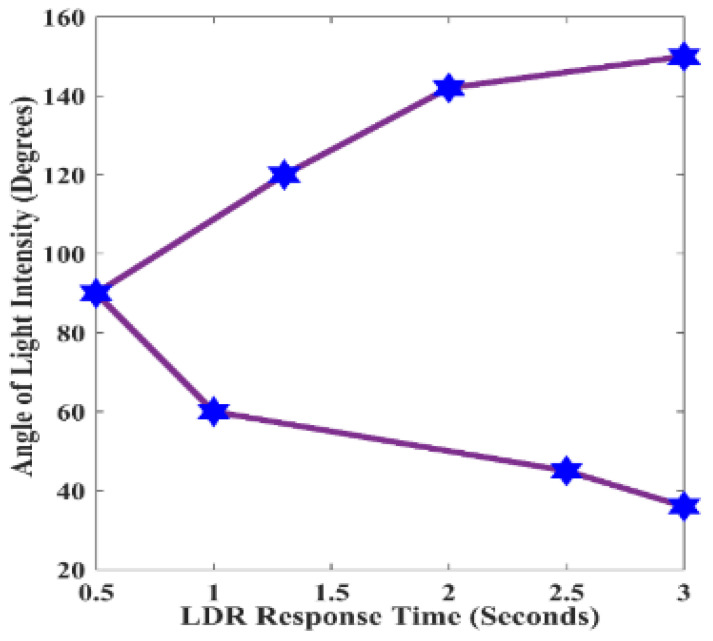
LDR response time and angle of the light source.

Figure 9 further demonstrates the response time of the LDR sensor at various angle positions, providing valuable insights into its sensitivity and reaction time to different high beam angles. At a 90-degree angle position, the LDR sensor exhibits maximum luminous intensity detection and the fastest response time, indicating its highest sensitivity and responsiveness to light changes when the source is directly in front of it. This suggests that the LDR sensor reacts most swiftly and is most sensitive to luminous intensity changes at this angle position. The observed trend depicted in Figure 9 shows a consistent decrease in response time as the angle of the high beam increases, indicating that the LDR sensor becomes quicker in detecting luminous intensity changes as the light source moves closer to the 90-degree angle position. Notably, at a 90-degree angle position, the LDR sensor exhibits its fastest response time of 0.5 s, highlighting its exceptional responsiveness when the high beam is perpendicular to its position. However, the data also indicates that beyond 90 degrees, the LDR sensor’s response time increases abruptly, indicating a reduced sensitivity to luminous intensity changes. For example, at an angle position of 152 degrees, the response time aligns with the time observed at 36 degrees, taking about 3 s to respond. The study results reveal that the LDR sensor’s response time is inversely proportional to the angle of the high beam. It is most sensitive and responsive at a 90-degree angle, while its responsiveness diminishes as the angle deviates from this perpendicular position. Understanding the characteristics of the LDR sensor’s response to high beams at different angle positions is crucial for designing effective intelligent headlight systems, particularly in optimizing the sensor’s positioning for the most effective light detection and responsiveness.

Having understood the characteristics of the LDR sensor, the next phase of the study integrated all the sensors proposed for this study to determine the feasibility of the proposed approach. To achieve this goal, a radar sensor and a buzzer were integrated into the intelligent headlight system. This was performed to distinguish the proposed intelligent headlight system from the conventional sensor-based intelligent headlights that employ ultrasonic sensors, which creates problems for the system.

The study’s aim to design an Arduino microcontroller-based intelligent headlight has proven to be feasible due to the successful simulation of the convoluted proposed intelligent headlight system. The key performance indicators (KPIs) set to assess the functionality of the proposed intelligent headlight were successfully met, confirming the feasibility and successful operation of the proposed intelligent headlight system.

### Validation

**Figure 10:**
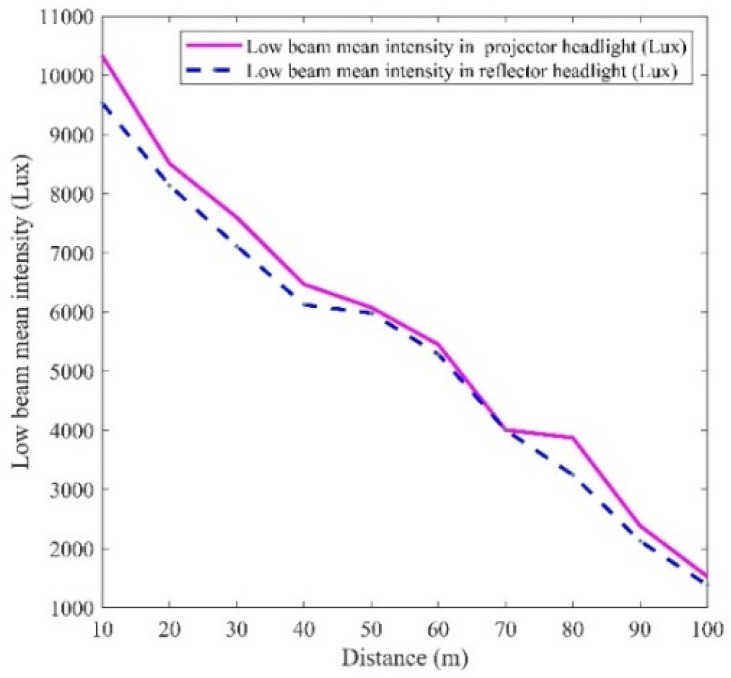
Low beam reference luminous intensity.

**Figure 11:**
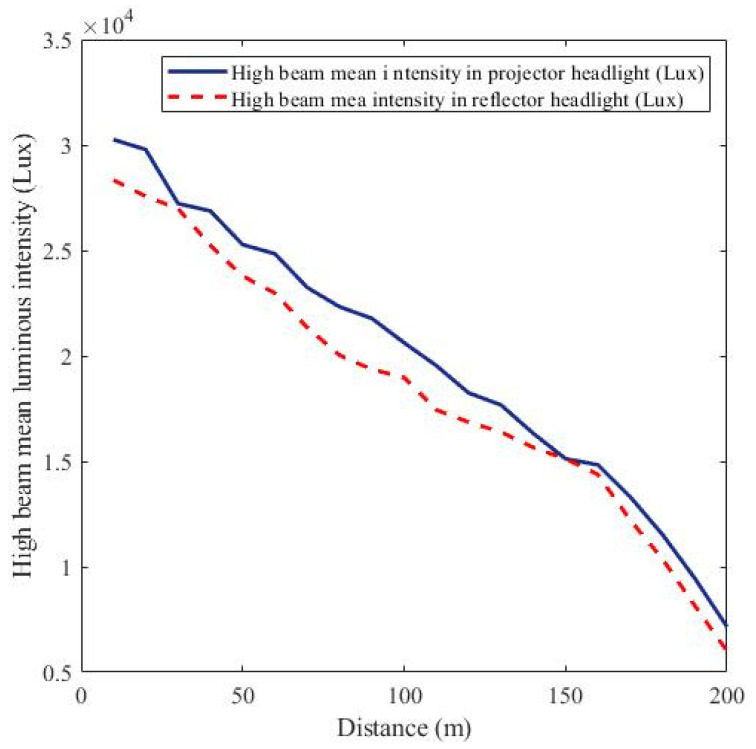
High beam reference luminous intensity.

In designing an efficient and effective intelligent headlight system using a sensor-based approach, determining a representative reference luminous intensity for the controller programming is crucial. This reference luminous intensity, stored in the controller, serves as a threshold for comparison, guiding the controller’s actions when the luminous intensity falls below or exceeds this preset value. This study established the reference luminous intensity by quantifying the luminous intensities produced by halogen, HID, and LED light sources in both projector and reflector headlight designs. The average intensities of these light sources were calculated for each headlight design. Using mathematical models referenced in Equations (3) and (6), the reference luminous intensities for the low and high beams were determined. The convergence point of the low beams in the reflector and projector headlights was selected as the low beam reference luminous intensity for the controller programming. Similarly, the convergence point of the high beams in both headlight designs was used to establish the high beam reference luminous intensity, as shown in Figure 10 and Figure 11. Figure 10 illustrates that at approximately 70 m from both the projector and reflector headlights, the two low beams converge with a luminous intensity of about 4000 lux. This finding supported the adoption of 4000 lux as the low beam reference luminous intensity for programming the controller for real-world applications. Likewise, the high beam reference luminous intensity was determined using Figure 11. As shown in Figure 11, the two high beams in both headlight designs converge at around 150 m from the headlights, with a luminous intensity of 16,000 lux. This led to the adoption of 16,000 lux as the reference luminous intensity for the high beam in the controller design at the practical implementation phase.

After a successful simulation to assess its feasibility, the proposed intelligent headlight system was implemented on an actual vehicle. To facilitate this implementation, the existing headlight connectors on the selected vehicle were replaced with new connectors suitable for integration with the microcontroller and other components of the intelligent headlight system. During the integration process, the high beam connections on both sides were linked to the Normally Closed terminal of the relay, while the low beams on both sides were connected to the Normally Open terminal of the relay. The Single SRD-05VDC-SL-C relay was selected for its reliability and compatibility with the Arduino UNO microcontroller, which typically outputs a relatively low-voltage signal. This relay effectively enables the microcontroller to control high-voltage components, such as the headlight in the actual implementation on the real vehicle and a Delphi radar sensor positioned behind the radiator for optimal performance. The Delphi radar sensor was chosen for its reliability and versatility, offering mid-range coverage of up to 60 m with a wide beam and long-range coverage extending to 174 m with a narrower beam.

**Figure 12:**
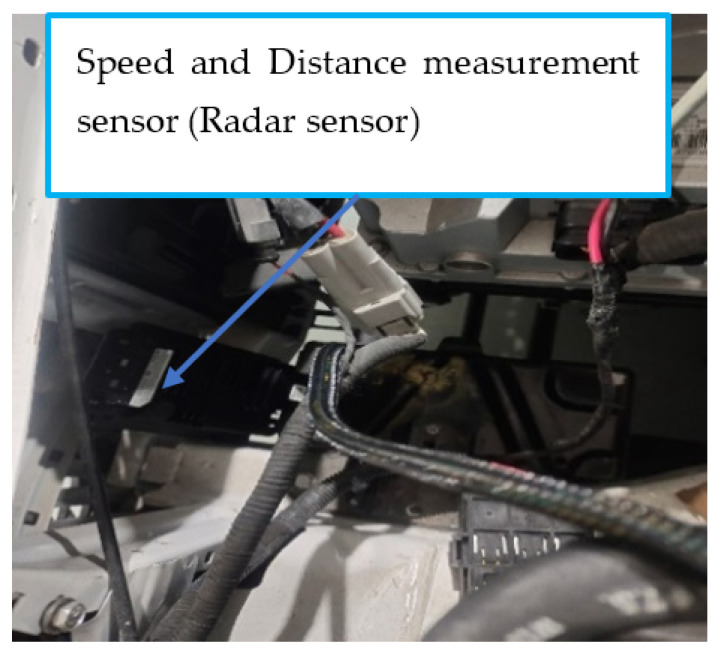
Location of the Radar sensor in the engine.

Additionally, it can discriminate and track up to 64 targets within the vehicle’s path. The radar sensor was positioned in the engine, as shown in Figure 12. Both the radar and LDR sensors were connected as input devices to the Arduino microcontroller, along with the alert system designed to notify the driver. The operation of the intelligent headlight system relies on comparing the output signals from the LDR and radar sensors to determine the appropriate headlight output. These components, along with the relay and Arduino microcontroller, were installed on the dashboard for convenient access and monitoring. The system automatically switches the headlight output from a high beam to a low beam when it detects a high luminous intensity above the reference luminous intensity of 16,000 lux from the opposite side of the road during nighttime driving. Implementing the proposed approach on a real vehicle further confirmed the feasibility of the Arduino microcontroller-based intelligent headlight system. The microcontroller was programmed to activate the relay and adjust the headlight output only when the radar sensor detects movement from a light-emitting object.

**Figure 13:**
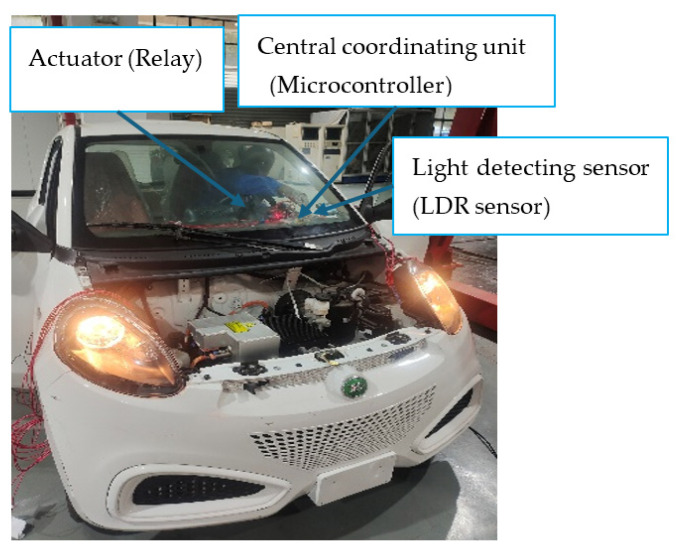
Headlight output in high beam.

Therefore, if the LDR sensor detects luminous intensity greater than 16,000 lux, indicating high beam luminous intensity, and the radar sensor detects movement, the headlight output is adjusted to a low beam. Conversely, as shown in Figure 13, the headlight output remains in the high beam state when the LDR sensor detects luminous intensity lower than the preset luminous intensity threshold of 16,000 lux.

**Figure 14:**
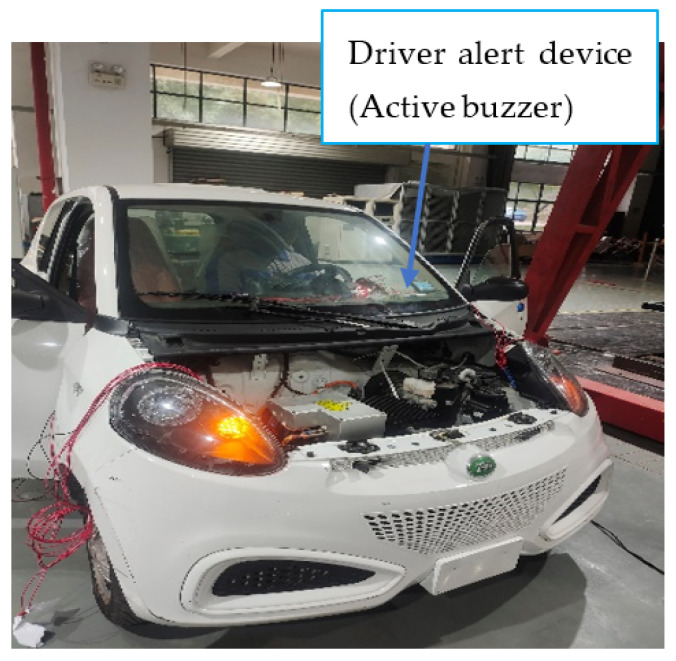
Headlight output in low beam.

The proposed intelligent headlight system is designed to automatically switch from high beam to low beam when the detected light intensity exceeds a set threshold of 16,000 lux. This switch is triggered by the LDR sensor, which detects oncoming vehicle headlights, enabling the system to reduce glare for other drivers. As part of this transition, a buzzer depicted in Figure 14 sounds to alert the driver that the headlight output has adjusted to a low beam. Figure 13 demonstrates the system’s response when the LDR sensor detects high luminous intensity and the radar sensor simultaneously detects an approaching vehicle. In such situations, the system promptly switches from high to low beam to avoid blinding oncoming drivers, thereby enhancing safety on highways.

**Figure 15:**
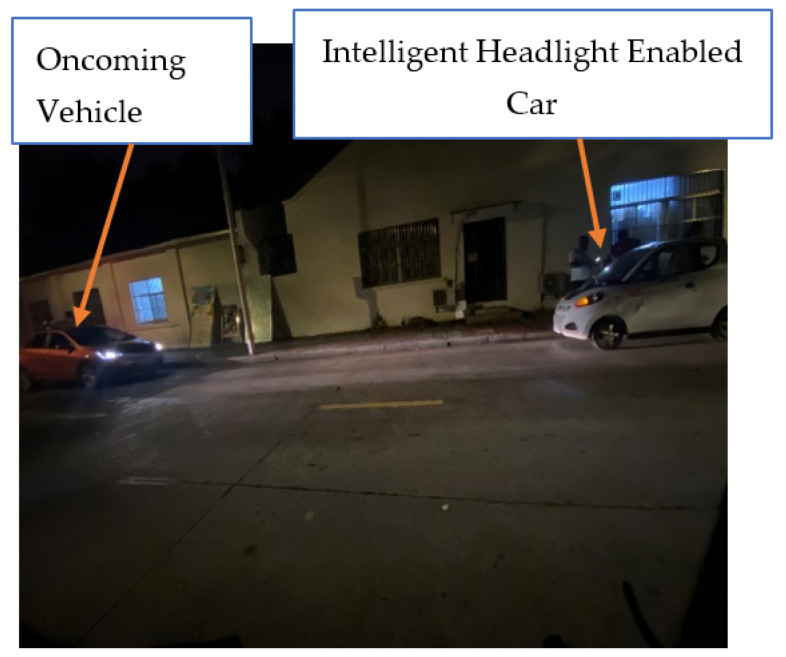
The intelligent headlight output switched to a low beam when the LDR sensor detected high beam luminous intensity.

To further verify the system’s effectiveness, a field test was conducted in a real-world environment. This test took place on a road featuring streetlights and in a residential area with illuminated buildings. Such a setting was chosen deliberately to test the system’s ability to differentiate between moving vehicles and stationary lights, ensuring the intelligent headlight system performs as intended. The field test results were highly promising, confirming the feasibility and reliability of implementing this Arduino microcontroller-based intelligent headlight control system. The system followed the programmed algorithms accurately, meeting all expectations. Figure 15 illustrates the system’s responsiveness during the field test: when a test vehicle positioned 10 m away activated its high beams towards the intelligent headlight-equipped vehicle, the system responded by immediately switching to a low beam. This prompt adjustment, as shown in Figure 15, highlights the system’s capability to effectively manage headlight intensity in response to real-time traffic conditions, supporting the viability of this intelligent headlight solution for enhancing road safety.

## 4. Conclusions

This study successfully designed and developed an Arduino microcontroller-based intelligent headlight system capable of distinguishing between streetlights, illuminated buildings, and vehicle headlight beams. By addressing the limitations of traditional sensor-based systems, the proposed solution introduced a radar sensor, which significantly enhanced the system’s ability to differentiate between moving and stationary lights, a key improvement over existing approaches. The system also incorporated an alert mechanism to inform drivers when their headlights transition from high beam to low beam, ensuring the driver’s focus remains on the road for a safer nighttime driving experience. Simulation results demonstrated an impressive result, particularly excelling when the approaching vehicle was at an angle of 90°. The radar sensor proved highly effective in both distance measurement and motion detection, validating its use in intelligent headlight systems. Furthermore, real-world testing on a vehicle confirmed that the system performed as anticipated, with the alert system providing an additional layer of safety not typically found in existing headlight technologies. While the proposed system showed great promise, the study did highlight some limitations, particularly the short-range and nonlinear characteristics of the LDR sensor and its susceptibility to bad adverse weather conditions, unlike the radar sensor employed for this study, which is immune to bad weather conditions. This makes LDR sensors unsuitable for widespread adoption in real-world scenarios. To improve future designs, it is recommended that sensors with more linear characteristics, such as photodiodes and phototransistors, be considered as alternatives to LDR sensors. The Arduino-based intelligent headlight system presents a cost-effective and practical solution that could be implemented across a wide range of vehicles, potentially making roads safer for nighttime driving. With further improvements, especially in sensor selection, the proposed system could serve as a viable alternative to the expensive machine learning-based headlight systems currently limited to high-end vehicles.

## Data Availability

The materials employed for this study have been listed in Section 2. The measurement results have also been presented, and the measuring instrument used for the experiment has been indicated, along with the measurement distances. For repeatability, any other author seeking to validate our results can utilize the same materials and methods applied in this study.
